# A new perspective on metformin therapy in type 1 diabetes

**DOI:** 10.1007/s00125-017-4364-6

**Published:** 2017-08-02

**Authors:** Rachel Livingstone, James G. Boyle, John R. Petrie, on behalf of The REMOVAL Study Team

**Affiliations:** 1 Institute of Cardiovascular and Medical Sciences, BHF Glasgow Cardiovascular Research Centre, University of Glasgow, 126 University Place, Glasgow, G12 8TA UK; 2 Glasgow Royal Infirmary, Glasgow, UK; 3 School of Medicine, University of Glasgow, Glasgow, UK

**Keywords:** Atherosclerosis, Cardiovascular, Carotid intima-media thickness, Cholesterol, Metformin, Review, Type 1 diabetes

## Abstract

Metformin is quite frequently used off-label in type 1 diabetes to limit insulin dose requirement. Guidelines recommend that it can improve glucose control in those who are overweight and obese but evidence in support of this is limited. Recently-published findings from the REducing with MetfOrmin Vascular Adverse Lesions (REMOVAL) trial suggest that metformin therapy in type 1 diabetes can reduce atherosclerosis progression, weight and LDL-cholesterol levels. This provides a new perspective on metformin therapy in type 1 diabetes and suggests a potential role for reducing the long-term risk of cardiovascular disease.

**Electronic supplementary material:**

The online version of this article (doi:10.1007/s00125-017-4364-6) contains peer-reviewed but unedited supplementary material, which is available to authorised users.

## Introduction

Over the last three decades, the Diabetes Control and Complications Trial (DCCT) and its Epidemiology of Diabetes Interventions and Complications (EDIC) post-randomisation follow-up have confirmed that the risk of microvascular and cardiovascular complications in type 1 diabetes can be reduced with intensive glucose control [1, 2]. However, despite modern insulin formulations administered by increasingly user-friendly and efficient modes of delivery, achieving and maintaining target blood glucose levels remains an elusive goal for many people affected by the condition [3].

One key barrier to achieving target blood glucose control is the risk and fear of hypoglycaemia. In the DCCT, rates of severe hypoglycaemia increased exponentially as HbA_1c_ approached target levels [1]. This problem is becoming more surmountable, at least for very motivated young individuals using continuous subcutaneous insulin delivery [4]. Another barrier is insulin-induced weight gain leading to insulin resistance and an associated escalating insulin dose requirement, increasing blood pressure and LDL-cholesterol levels [5].

The concept of adjunct therapy for type 1 diabetes has emerged in response to these challenges and is based on the notion that: (1) adding a simple (oral) preparation to insulin therapy might help to improve glycaemic control; and (2) such additional therapeutic agents might have effects independent of glucose lowering to reduce the risk of diabetes complications. The ideal adjunct therapy would, therefore, reduce insulin dose requirement, lower HbA_1c_ without increasing the risk of hypoglycaemia, reduce weight, and have direct effects to reduce the risk of cardiovascular disease and improve life expectancy [6, 7].

Diabetologists over previous decades have thought that metformin might be such a therapy, potentially mirroring its effects in type 2 diabetes in those with type 1 diabetes. In this review, we chart the evolution of this idea from small studies in the 1980s up to the recent REducing with MetfOrmin Vascular Adverse Lesions (REMOVAL) study, the largest trial to date of metformin in the management of type 1 diabetes [8, 9].

## Metformin

As discussed by Sanchez-Rangel and Inzucchi in this issue of *Diabetologia* [10], metformin hydrochloride is a simple and inexpensive biguanide molecule that is currently the first-line oral glucose-lowering agent in international guidelines for the management of type 2 diabetes [11, 12]. Its widespread uptake worldwide was driven by the UK Prospective Diabetes Study (UKPDS), published in 1998 [13]. Prior to the UKPDS, metformin was usually reserved for obese individuals and used with caution because of its similarity to another biguanide, phenformin, that was withdrawn from use in 1977 owing to concerns regarding a potential increased risk of lactic acidosis. Metformin was not withdrawn in the UK but was unavailable in the USA between 1977 and 1995. Key findings from the metformin substudy of the UKPDS were that obese participants with type 2 diabetes gained less weight than those on other oral therapies, had lower rates of hypoglycaemia and had a 33% reduction in the risk of myocardial infarction [13, 14].

Perhaps because of such compelling evidence in type 2 diabetes, off-label use of metformin in type 1 diabetes is quite common in clinical practice. In a 2016 extract of population data from Scotland, UK, 15% of adults with type 1 diabetes had received at least one prescription for metformin and 8% were using it currently [9]. If practice is similar elsewhere, it is likely that metformin is currently prescribed for thousands of people with type 1 diabetes worldwide. In France, in the form of metformin embonate, it has held a product license for use in type 1 diabetes since 1996. However, as discussed below, clinical evidence to guide this practice has been lacking.

## Early studies: the 1980s

A small double-blind placebo-controlled crossover trial conducted in France in the mid 1980s reported an improvement in euglycaemic–hyperinsulinaemic clamp-assessed insulin sensitivity when metformin was added to insulin therapy for 7 days in ten non-obese people with type 1 diabetes [15]. Two years later, in 1987, the late Harry Keen presented an abstract at the EASD Annual Meeting describing a double-blind, placebo-controlled crossover trial in eight people with type 1 diabetes, conducted over 3 weeks, in which no change in fasting glucose, body weight or insulin dose requirement was detected with metformin use, despite a significant improvement in seven-point capillary glucose profile [16].

## Small randomised trials: the 2000s

The early studies described above did not ignite enthusiasm for research on metformin in type 1 diabetes in the 1990s. It was more than a decade later, after the publication of the UKPDS, that a research group from Colorado (USA) presented an abstract at the ADA Meeting in 2000 describing a double-blind placebo-controlled trial in which 80 adolescent participants with type 1 diabetes achieved non-sustained improvements in HbA_1c_ and weight at 3 months with metformin use, reverting to baseline by 6 months [17]. The full paper was not published until 15 years later [18].

In 2002, a group in New Hampshire (USA) reported a double-blind placebo-controlled trial, in which 62 adults with type 1 diabetes were randomised to receive metformin or placebo, showing a reduction in insulin dose requirement without an improvement in HbA_1c_ when metformin was added to continuous subcutaneous insulin infusion therapy for 6 months [19]. The following year (2003), two double-blind placebo-controlled trials, one carried out in Canada [20] and the other in Sweden [21], each randomising 30 adolescent individuals with type 1 diabetes, reported improvements in HbA_1c_ after 3 months of metformin use (by 0.6% [6.6 mmol/mol] and 0.9% [9.9 mmol/mol], respectively); only the Canadian study demonstrated a significant reduction in insulin dose requirement and fasting glucose [20] and neither detected a difference in weight or in insulin sensitivity [20, 21]. A small study in the UK, published in 2006, had similar findings to the Canadian study [22].

Other small non-randomised studies (for example [23]) continued to appear as the decade progressed, but there was clearly a need for studies in larger groups of individuals if the field was to move forward. In 2008, Lund et al published a double-blind study placebo-controlled trial in which 100 individuals with type 1 diabetes and suboptimal glycaemic control were randomised to metformin or placebo for 1 year at the Steno Diabetes Center (Copenhagen, Denmark). There was no improvement in HbA_1c_ with metformin, but reductions in insulin dose requirement, weight and LDL-cholesterol (by 5.7 U, 1.74 kg and 0·3 mmol/l, respectively) were observed [24, 25]. The reduction in LDL-cholesterol did not diminish following adjustment for a statistically significant imbalance in concomitant statin use (used in 49% of the metformin group and 27% of the placebo group) [25]. Around 40% of participants in both groups reported gastrointestinal adverse effects during the trial but very few discontinued treatment. The number of individuals with at least one episode of severe hypoglycaemia was numerically, but not statistically, higher with metformin compared with placebo but the rate of severe hypoglycaemia complicated by coma (*n* = 6 with metformin; *n* = 1 with placebo) approached statistical significance.

## Systematic review: 2010

In our systematic review and meta-analysis of these trials [26], the study by Lund et al [24] contributed more than half of the 192.8 patient-years available for analysis. We highlighted the paucity of evidence from only nine small randomised, double-blind trials, only five of which could be summarised in a meta-analysis because of heterogeneity. We noted a significant reduction in insulin dose requirement (6.6 U/day, *p* < 0.001) with metformin, and weight reduction in some trials, but no consistent evidence for HbA_1c_ reduction. We found no information on cardiovascular outcomes, whether clinical or surrogate but, in view of the findings by Lund et al, we flagged LDL-cholesterol as an outcome worthy of further study.

Later that year, the UK National Institute for Health and Care Excellence (NICE) replicated our meta-analysis and went on to recommend metformin for adults with type 1 diabetes and a BMI ≥25 kg/m^2^ who ‘want to improve glucose control while minimising their effective insulin dose’ [27]. The ‘recommendations for research’ included further studies on metformin in type 1 diabetes, although outcomes of interest were not specified.

The ADA followed, stating that ‘adding metformin to insulin therapy may reduce insulin requirements and improve metabolic control in overweight/obese patients with poorly controlled type 1 diabetes’ [28]. Given that around 50% of people with type 1 diabetes are either obese or overweight in contemporary cohorts [5], and more than 30% have poor glycaemic control [3], following these recommendations would have led to a sharp rise in metformin prescribing in type 1 diabetes.

## Lessons from type 2 diabetes?

Types 1 and 2 diabetes are conditions with very different aetiology and pathogenesis, but many people with long-duration type 2 diabetes require insulin treatment, and the risk of cardiovascular disease is high in both conditions. A small double-blind placebo-controlled crossover trial conducted in the Netherlands, published in 2000, demonstrated reductions in HbA_1c_, weight and insulin dose requirement when metformin was added to treatment for 5 months in insulin-treated type 2 diabetes [29]. This was followed, in 2009, by the Hyperinsulinaemia: the Outcome of its Metabolic Effects (HOME) trial, a double-blind placebo-controlled trial conducted by a different group in the Netherlands. This study was carried out on a high-risk population with type 2 diabetes and, thus, had sufficient statistical power to specify clinical microvascular and cardiovascular outcomes [30]. In 390 insulin-treated individuals with type 2 diabetes studied over 4.3 years, there was a striking 40% reduction in cardiovascular events, a pre-specified secondary outcome, in those randomised to metformin. HbA_1c_, insulin dose requirement and weight gain were also reduced (by 0.4%, 20 U/day and 3 kg, respectively). That metformin could provide cardiovascular protection in insulin-treated individuals with type 2 diabetes provided proof of concept, albeit by extrapolation, for a similar effect in type 1 diabetes. In terms of mechanism, cholesterol was not lowered but improvements were observed in several biomarkers of endothelial dysfunction [30, 31].

## Larger trials in type 1 diabetes: 2010 to present

### The Type 1 Diabetes (T1D) Exchange study

On the basis of supportive data with continuing uncertainty, in 2010 the US type 1 diabetes charity JDRF decided to prioritise funding for metformin as adjunct therapy in type 1 diabetes. The first of the multicentre trials to emerge (in 2016) was a double-blind, placebo-controlled randomised clinical trial in 140 overweight and obese adolescent individuals with poor glycaemic control (mean HbA_1c_, 8.8% [73 mmol/mol]) and high insulin dose requirements (mean, 1.1 U/kg) [32]. HbA_1c_ was reduced at 3 months with metformin (by 0.3% [3.3 mmol/mol]) but this was not sustained at the end of the 6 month trial (similar findings to those previously reported in 2000 by the Colorado group [17]). As for other pre-specified outcomes, insulin dose requirement was reduced by 25% from baseline in 23% of participants taking metformin (compared with 1% in the placebo group) and BMI was reduced by 10% or more in 24% of participants taking metformin (compared with 7% in the placebo group) [33]. No changes were observed in cholesterol levels. The researchers concluded that their results did not support prescribing metformin to overweight adolescent individuals with type 1 diabetes to improve glycaemic control and, by implication, did not support the guideline recommendations, at least in younger individuals.

### The REMOVAL study

The REMOVAL study was another multicentre trial funded by JDRF. Led by one of the current authors (JRP), it was an international effort that aimed to address the lack of cardiovascular data in the area of metformin use in type 1 diabetes by conducting a double-blind, placebo-controlled trial to test whether 3 years of metformin treatment (1000 mg twice daily) added to titrated insulin therapy (towards target HbA_1c_, 7.0% [53.0 mmol/mol]) reduces progression of atherosclerosis in adults aged 40 years or older with confirmed type 1 diabetes and three or more cardiovascular risk factors [8, 9].

Carotid artery intima-media thickness (cIMT) was selected as a surrogate outcome for atherosclerotic cardiovascular disease, since: it predicts cardiovascular events in the general population [33]; and it was reduced over 6 years of the EDIC [34] follow-up study in participants with type 1 diabetes after 6.5 years of intensive glucose control in the DCCT and this was followed by improved cardiovascular outcomes over 30 years [2]. Prior to REMOVAL, small studies had reported a reduction of cIMT with metformin use in the metabolic syndrome and type 2 diabetes [35, 36]. However, while REMOVAL was under way, the Carotid Atherosclerosis: MEtformin for insulin ResistAnce (CAMERA) trial reported no impact of 18 months of metformin treatment on cIMT in individuals without diabetes but with established coronary heart disease [37], and the Copenhagen Insulin and Metformin Therapy (CIMT) trial reported no reduction in cIMT progression in insulin-treated people with type 2 diabetes [38].

In the REMOVAL study, progression of the primary outcome, mean far wall cIMT, was not significantly reduced with metformin therapy. Mean cIMT is often used in studies of people without diabetes to reduce variability as it excludes individual readings of intima-media thickness ≥1.5 mm and plaque (in keeping with the Mannheim Consensus [39]). However, the tertiary outcome, maximal far wall cIMT, pre-specified in REMOVAL because of its use in the DCCT/EDIC study, was reduced by metformin by twice as much as in the EDIC study over less than half the period of follow-up, despite more than 80% of participants being on statins [9]. Maximal cIMT is a measure of more advanced stages of atherosclerotic disease, including focal thickening and plaque [40]. This was an exciting and positive finding but it is premature to conclude that the effect of metformin on cIMT in the REMOVAL study might translate into clinical outcomes, as the contribution of reduced cIMT progression, per se, to lowered cardiovascular disease outcome rates independent of blood glucose levels has not been formally explored in the DCCT/EDIC.

Along with a change in vascular structure, results of the REMOVAL study demonstrated a sustained reduction in weight with metformin (by 1.2 kg), as well as modest reductions in insulin dose requirement (by about 2 U/day from the 6-month time point onwards) and LDL-cholesterol (by 0.13 mmol/l), despite the high prevalence of statin use [9] (see Table 1). There was no increase in hypoglycaemia and no effect of metformin on reactive hyperaemia index, a measure of small vessel endothelial function. Estimated GFR was acutely increased upon initiation of metformin (by 4 ml min^−1^ 1.73m^−2^), an unexpected finding that requires further investigation, although it is in keeping with some other recent evidence [41]. Around a quarter of individuals (twice the rate of those taking placebo) discontinued metformin over 3 years, suggesting that about one in eight had genuine intolerance with the majority being attributable to gastrointestinal adverse effects. Biochemical vitamin B12 deficiency was more than doubled over 3 years with metformin use (12%) vs placebo (5%). These findings contribute to a body of evidence that treatment with metformin reduces vitamin B12 concentration, suggesting monitoring during long-term use [28], particularly in type 1 diabetes, in which there is associated risk of gastroparesis, pernicious anaemia and coeliac disease.

**Table 1:** Summary of REMOVAL study outcomes

Outcomes	Baseline (mean ± SD)	Difference or ratio (metformin vs placebo)	Main effect (*p* value)	Treatment-by-visit interaction^a^ (*p* value)	Effect of metformin over 3 years: clinical interpretation
Primary outcome					
Mean cIMT(mm)	0.782 ± 0.162	−0.005	0.166	−	No significant reduction in progression of arteriosclerosis
Secondary outcomes					
HbA_1c_ (%)	8.1 ± 0.8	−0.13	0.006	0.016	Reduction at 3 months by 0.24% (2.6 mmol/mol) (*p* < 0.0001) but not sustained thereafter
HbA_1c_ (mmol/mol)	64.0 ± 9.0	−1.4	0.006	0.016	
LDL-cholesterol (mmol/l)	2.20 ± 0.71	−0.13	0.012	0.310	Sustained reduction by 0.13 mmol/l
eGFR (ml min^−1^ [1.73 m]^−2^)	92.0 ± 21.2	+4.0	<0.0001	0.662	Increased 4 ml min^−1^ (1.73 m)^−2^ at 3 months, then declined in parallel with placebo; requires further investigation
Retinopathy (two-step change from baseline)	−	0.76 (OR)	0.568	−	No effect
Weight (kg)	84.0 ± 14.7	−1.17	<0.0001	0.274	Sustained reduction by 1.17 kg
Insulin dose (U/kg)	0.65 ± 0.28	−0.005	0.545	0.002	After 6 months, reduced by 2 U/day (*p* = 0.045; post hoc analysis)
Endothelial function (reactive hyperaemia index; AU)	2.26 ± 0.74	−0.06	0.302	0.566	No significant change
Tertiary outcomes					
Severe hypoglycaemia (per patient year)	0.16	1.23 (IRR)	0.442	−	No significant change
Treatment satisfaction	31.77 ± 3.94	−0.12	0.668	0.629	No significant change
Maximal common cIMT(mm)	0.918 ± 0.196	−0.013	0.0093	−	Significant reduction in progression of atherosclerosis
Occurrence of vitamin B12 deficiency (<150 pmol/l)	−	2.76 (HR)	0.0094	−	Risk of vitamin B12 deficiency more than doubled vs placebo

Data were analysed by ANCOVA other than for carotid outcomes (repeated measures regression), retinopathy (logistic regression), hypoglycaemia (negative binomial regression) and vitamin B12 (Cox proportional hazards)

All analyses were pre-specified unless otherwise stated

^a^Presented for data analysed by ANCOVA only

AU, arbitrary units; eGFR, estimated GFR; IRR, incidence rate ratio

As in other studies of type 1 diabetes and metformin, HbA_1c_ was only transiently improved by metformin and reverted to baseline over the first 6 months of use, probably as insulin doses were down-titrated by patients in an effort to avoid hypoglycaemia [9]. The effect of metformin on maximal cIMT was, therefore, consistent with an anti-atherosclerotic effect independent of glucose lowering. Candidate mechanisms include those mediated by activation of AMP-activated kinase (AMPK) [42], for example, inhibition of proinflammatory pathways in perivascular adipose tissue [43], inhibition of monocyte-to-macrophage differentiation in vascular tissues [44] or improvement in aspects of endothelial function [31, 45]. Alternatively, metformin can inhibit AGE formation by a pathway independent of AMPK [46]. The contrasting results between the REMOVAL study and the CAMERA study [37] suggest that an effect of metformin on atherosclerosis progression may be specific to diabetes. In the CIMT trial, as acknowledged by the authors, the lack of an effect of metformin on cIMT in type 2 diabetes may have been due to a lack of statistical power [36].
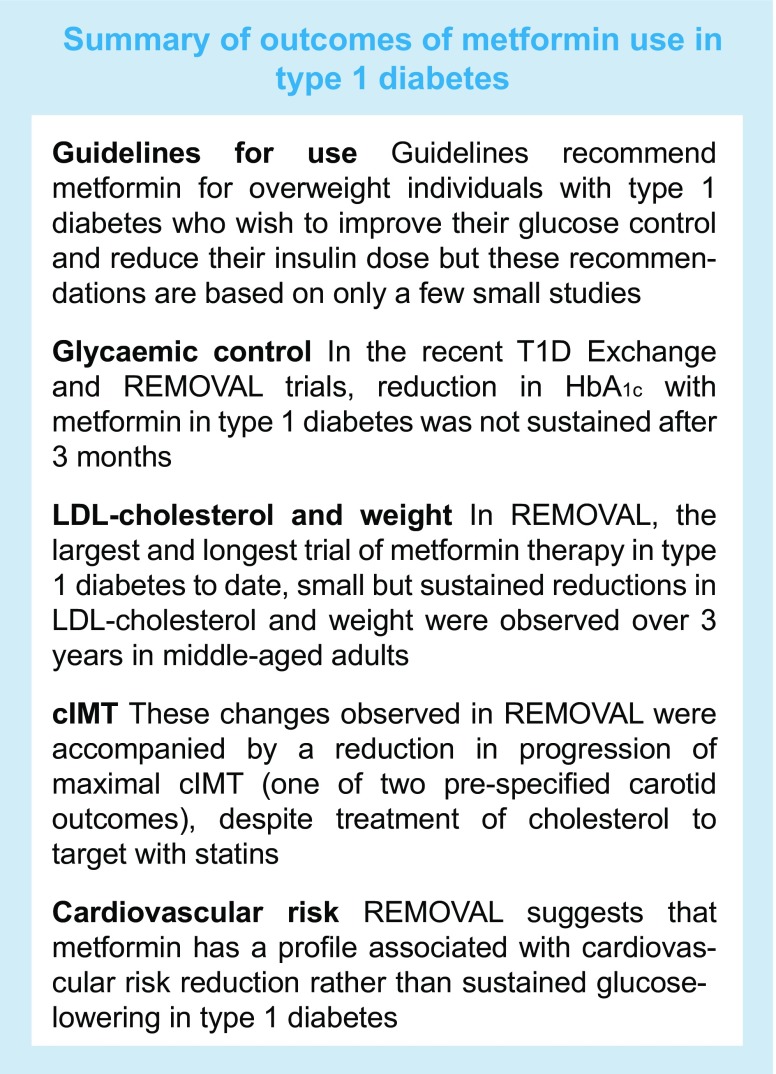


## Summary and conclusions

The recent larger trials provide a new perspective on the use of metformin in type 1 diabetes. Although there is evidence that metformin can limit insulin dose requirement, there is little evidence to support the recommendation by current guidelines that it can help to improve glucose control in individuals with type 1 diabetes who are overweight or obese.

Ideally there should be a cardiovascular outcome trial of metformin in type 1 diabetes but this would involve studying several thousand individuals over at least 5 years, currently considered too expensive by the major funding bodies and not a priority for the pharmaceutical industry. This is a striking contrast to the situation in type 2 diabetes: no outcome-based trials of any intervention have been conducted in type 1 diabetes to date, with the exception of the DCCT and its EDIC follow-up study.

Until an outcome-based trial is completed, clinicians and people with type 1 diabetes will have to decide whether metformin will be of benefit on the basis of existing evidence. The results of the REMOVAL study suggest that metformin can reduce weight and LDL-cholesterol and might reduce atherosclerosis progression, over 3 years in middle-aged people with long-duration type 1 diabetes already treated with antihypertensive agents and statins. These data suggest wider off-label use to improve CVD risk management in type 1 diabetes.

## Electronic supplementary material

ESM The Removal Study Team (PDF 26.9 kb)

## Abbreviations

AMPK: AMP-activated kinase
CAMERA: Carotid Atherosclerosis: MEtformin for insulin ResistAnce
cIMT: Carotid artery intima-media thickness
CIMT: Copenhagen Insulin Metformin Therapy
DCCT: Diabetes Control and Complications Trial
EDIC: Epidemiology of Diabetes Interventions and Complications
NICE: National Institute for Health and Care Excellence
REMOVAL: REducing with MetfOrmin Vascular Adverse Lesions
UKPDS: UK Prospective Diabetes Study
