# Supplementary material for: A new perspective on metformin therapy in type 1 diabetes
Source: Diabetologia. 2017 Aug 2;60(9):1594–600. doi: 10.1007/s00125-017-4364-6 (PMC5552844; doi:10.1007/s00125-017-4364-6)
Supplement: Supplementary file 1 — (PDF 26.9 kb) [file 125_2017_4364_MOESM1_ESM.pdf]

The REMOVAL Study Team

Steering Committee

J Petrie (Chair and Chief Investigator; University of Glasgow, UK); H Colhoun (Deputy Chief Investigator; University of Dundee, UK); N Chaturvedi (University College London, UK), I Ford (University of Glasgow, UK); I Hramiak[a] (University of Western Ontario, ON, Canada); A Hughes (University College London, UK); A Jenkins[a] (University of Sydney, NSW, Australia); B Klein (University of Wisconsin, WI, USA); R Klein (University of Wisconsin, WI, USA); T Ooi (The Ottawa Hospital, ON, Canada); P Rossing[a] (Steno Diabetes Center, Denmark); N Sattar (University of Glasgow, UK); C Stehouwer[a] (University of Maastricht, Netherlands). H Nickerson, O Lou, S Dutta (non-voting) (JDRF representatives, New York, NY, USA). Jonathan Haw (patient representative, London, UK; deceased); C Anderson (patient representative, Glasgow, UK).

[a]Also National Principal Investigator

Trial coordination

Robertson Centre for Biostatistics, University of Glasgow, UK: I Ford[b], S Kean, E Thomson, L Gillespie, J Gibb, N Greenlaw. Robarts Research Institute (ON, Canada): I Hramiak[b], S Tereschyn. NHMRC Clinical Trials Centre (Sydney, NSW, Australia): A Keech[b], A Jenkins[b]. Carotid Reading Centre (University College London, UK): N Chaturvedi[b], A Hughes[b], K March, S Williams, E Coady, T Tillin. Carotid External Quality Assurance (Julius Centre for Health Sciences, Utrecht, Netherlands): M Bots[b]. Retinal Grading Centre (University of Wisconsin, WI, USA): R Klein[b], B Klein[b], J Dreyer, T Jan. ENDOPAT Centre (Itamar Medical, Israel): Koby Sheffy[b], Ravit Lusky, Shlomit Peleg. ENDOPAT Committee: J Petrie[b] (Glasgow,

UK), H Colhoun (Dundee, UK), A Shore (Exeter, UK), D Carty, C Delles (Glasgow, UK). Data Monitoring Committee: P Donnan[b] (Dundee, UK), M Witham (Dundee, UK), A Adler (Cambridge, UK), E Lonn (Toronto, ON, Canada), P Rauchhaus (DMC Statistician, Dundee, UK). Glycaemia Committee: I Hramiak[b] (Ontario, ON, Canada), R Lindsay (Glasgow, UK), M Brouwers (Maastricht, The Netherlands). Project Management Unit (NHS Glasgow, UK): J Van-Melckebeke[b], L Gillespie, T Hamill, L Cuthbertson, A Murray, L Jolly, E Miller. Biomarker Laboratory (University of Glasgow, UK): N Sattar[b], P Welsh[b]. Biorepository (NHS Glasgow, UK): J Hair[b], A Bell; Drug Supply Management (NHS Glasgow, UK): S Carmichael[b], E Douglas, P Surtees. Pharmacovigilance (NHS Glasgow, UK): E Dinnett[b]: J Allan, S Kean. Data Monitoring (NHS Glasgow, UK): C Watson[b], M McLaughlin, G Brindley, E Smillie. Financial Management: D Motherwell[b], S MacDonald (Glasgow). Contracts and Agreements: P Ellis[b], D Stuart (University of Glasgow, UK); M Travers[b] (NHS Glasgow, UK). Scottish Diabetes Research Network: S Brearley[b], L Greig. Patient representative: J Haw (London, UK; deceased).

[b] Lead investigator or collaborator

Recruiting Centres and Site Staff

Australia Melbourne (Royal Melbourne Hospital, VIC): P Colman (PI), A Nankervis, S Forulanos; D West; S Vaughan, M Bjorasen; J Donlon[c]. Melbourne (St Vincent's Hospital, VIC): D O'Neal (PI), J Horsburgh, H Pater, S Kent, J Vrazas. Sydney (Royal Prince Alfred Hospital, NSW): S Twigg (PI), G Fulcher, R Denner, A Piotrowicz, A Coy, H Pater, A Januszewski[c].

Canada London, ON: I Hramiak (PI), T Paul, C McDonald, S Tereschyn, N Schmidt, M Weingert, M Weingertc, H Heardc, S Burkec. Ottawa, ON: TC Ooi (PI), H Lochnan (Co-PI),

A Sorisky, E Keely, J Malcolm, J Maranger, C Favreau, S Pethericke, K Bolesc.

Denmark Steno Diabetes Center (Gentofte): P Rossing (PI), TW Hansen, B Hemmingsenc.

England Bristol (Bristol Royal Infirmary): N Thorogood (PI), K Green, T Robinson[c]. Durham (University Hospital): K Abouglilia (PI), D Nayman, C Miller[c]. Exeter (Royal Devon and Exeter Hospital): R Warren (PI), K Aizawa[c]; Gloucester (Gloucestershire Royal Hospital): Dr M Balasubramani (PI), S Toth, K Harvey[c], G Birch[c]. Hull (Michael White Centre for Diabetes): T Sathyapalan (PI), A James, Z Javed[c]. Liverpool (Aintree University Hospital): J Wilding (PI), B Martin, S Birch, A Wilcox[c], N Watson[c]. London (St Mary's Hospital): N Oliver (PI), N Jugnee, K March[c]. Manchester (Central Manchester University Hospitals): M Rutter (PI), T Turgut (Co-PI), A Shaju, S Yau, S Subin[c]. Newcastle (Royal Victoria Infirmary): M Walker (PI), D Wake, C Miller[c]; Plymouth (Derriford Hospital): A Millward[a] (PI), P Chong (PI), M Hibbert, J George[c].

The Netherlands Maastricht University Medical Centre: Professor Coen Stehouwer (PI), MC Brouwers (Co-PI), N Schaper, J Pinxt, J op het Roodt[c].

Scotland Aberdeen (Aberdeen Royal Infirmary): S Phillips (PI), L Murray, Linda Sleigh[c]. Ayr (Ayr Hospital): A Collier/ LE Sit (PIs), K Allan, J Cook, K Campbell[c], L Hodge[c]. Dundee (Ninewells Hospital): G Leese, G Reekie, K Shields[c]. Edinburgh (Royal Infirmary): A Jaap (PI), A Sudworth, A White[c]. Edinburgh (Western General): J McKnight (PI), L Steven, A White[c]; Glasgow (Stobhill Hospital): G McKay (PI), A Llano (deputy-PI), G Currie, E Lennon, J Johnstone, K Shields[c].

[a]Also National Principal Investigator
[c]Sonographer
PI, Principal investigator
